# Brachialis Muscle Activity Can Be Measured With Surface Electromyography: A Comparative Study Using Surface and Fine-Wire Electrodes

**DOI:** 10.3389/fphys.2021.809422

**Published:** 2021-12-23

**Authors:** Shota Date, Hiroshi Kurumadani, Yuko Nakashima, Yosuke Ishii, Akio Ueda, Kazuya Kurauchi, Toru Sunagawa

**Affiliations:** ^1^Laboratory of Analysis and Control of Upper Extremity Function, Graduate School of Biomedical and Health Sciences, Hiroshima University, Hiroshima, Japan; ^2^Laboratory of Musculoskeletal Ultrasound in Medicine, Graduate School of Biomedical and Health Sciences, Hiroshima University, Hiroshima, Japan; ^3^Department of Biomechanics, Graduate School of Biomedical and Health Sciences, Hiroshima University, Hiroshima, Japan

**Keywords:** electromyelography, brachialis muscle, surface electrode, elbow flexors, biceps brachii muscle, fine-wire electrode

## Abstract

Muscle activities of the elbow flexors, especially the brachialis muscle (BR), have been measured with intramuscular electromyography (EMG) using the fine-wire electrodes. It remains unclear whether BR activity can be assessed using surface EMG. The purpose of this study was to compare the EMG patterns of the BR activity recorded during elbow flexion using surface and fine-wire electrodes and to determine whether surface EMG can accurately measure the BR activity. Six healthy men were asked to perform two tasks—a maximum isometric voluntary contractions (MVICs) task and an isotonic elbow-flexion task without lifting any weight. The surface and intramuscular EMG were simultaneously recorded from the BR and the long and short heads of the biceps brachii muscle (BBLH and BBSH, respectively). The locations of the muscles were identified and marked under ultrasonographic guidance. The peak cross-correlation coefficients between the EMG signals during the MVICs task were calculated. For the isotonic elbow-flexion task, the EMG patterns for activities of each muscle were compared between the surface and the fine-wire electrodes. All cross-correlation coefficients between the surface EMG signals from the muscles were lower than 0.3. Furthermore, the EMG patterns of the BR activity were not significantly different between the surface and the fine-wire electrodes. The BR has different EMG pattern from the BBLH and the BBSH. The BR activity, conventionally measured with intramuscular EMG, can be accurately accessed with surface EMG during elbow flexion performed without lifting any weight, independent from the BBLH and BBSH activities.

## Introduction

Elbow flexors consist of the brachialis (BR), biceps brachii (BB), and brachioradialis muscles (Murray et al., 2000). Previous studies have shown that the BR has the greatest contribution to elbow flexion torque/force (An et al., 1981; Kawakami et al., 1994; Murray et al., 2000; Genet et al., 2017). The BR also plays an important role in clinical practice such as for reacquiring the function of elbow flexion after reinnervation surgery following brachial plexus injury and post-stroke (Mackinnon et al., 2005; Li et al., 2007; Genet et al., 2017).

The BR activity has conventionally been investigated using intramuscular electromyography (EMG) with fine-wire electrodes (Basmajian and Latif, 1957; Buchanan et al., 1986; Hodges et al., 2003; Naito, 2004; Rudroff et al., 2008). Although intramuscular EMG has the advantage of being suitable for detecting individual muscle activity and minimizing crosstalk (Perry et al., 1981; Luca, 1997; Onishi et al., 2000), it is highly invasive, requires specialized expertise for electrode insertion (Õunpuu et al., 1997), and does not represent the electrical activity of the whole muscle (Bogey et al., 2000). In contrast, surface EMG is non-invasive, easy to perform, less uncomfortable for participants, and allows recording of the muscle’s electrical activity over a large area (Bogey et al., 2000; Knox et al., 2021). A recent study showed that surface EMG could measure the BR activity independent from that of the BB (Staudenmann and Taube, 2015); however, it did not validate the location of the elbow flexors, and the measured BR activity could have had interferences from the adjacent muscle activities. Furthermore, there have been no studies that have compared the EMG patterns of the BR activity measured using surface and fine-wire electrodes. Therefore, it remains unclear whether the BR activity can be measured using surface EMG, independent of the adjacent synergistic muscles.

In this study, we compared the EMG patterns of the BR activity obtained using surface and fine-wire electrodes and determined whether surface EMG can accurately measure the BR activity. We hypothesized that there would be no differences between the EMG patterns obtained when using surface and fine-wire electrodes, and that the EMG pattern of the BR activity was significantly different from that of the BB activity.

## Materials and Methods

### Subjects

Six male volunteers (average age ± standard deviation, 36.5 ± 15.1 years) participated in this study. The participants were healthy, had no history of motor and sensory dysfunction, and had no limitations in the range of motion of the elbow joint. All participants provided written informed consent before participating in the study. This study was approved by our institutional ethics review board and was performed in accordance with the Declaration of Helsinki.

### Experimental Task

The participants were asked to perform two tasks in a seated position using their non-dominant upper limb. The first task, which was aimed to examine whether surface EMG recorded from the BR was independent of the activity of other synergistic muscles, had the participants performed maximum voluntary isometric contractions (MVICs) of the elbow flexors. Participants were required to perform MVICs with the elbow flexed at 90° and the forearm supinated to 90°. Three repetitions of MVICs were performed by each participant, and each MVIC was maintained for 3 s. The second task required the participants to perform isotonic elbow flexion at three different forearm positions: supination, neutral, and pronation. This elbow-flexion task was performed without lifting any weight. The movement was repeated seven times for each forearm position. The time taken to achieve full flexion from full extension was approximately 1 s. Simultaneously with the EMG recordings, the kinematics of the elbow movements were recorded using a video camera to identify the elbow joint movement.

### Intramuscular and Surface Electromyography

**FIGURE 1:**
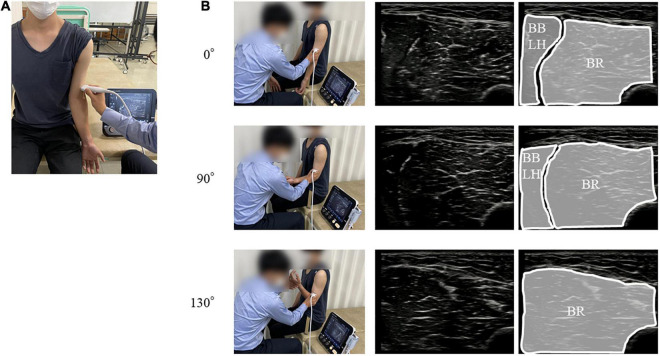
Position and orientation of the ultrasound probe. **(A)** The position of probe for the BR. **(B)** The upper, middle, and lower panels represent the position of probe for the BR and ultrasound images of the BR at the 0°, 90°, and 130° elbow flexions, respectively. BR, brachialis muscle.

**FIGURE 2:**
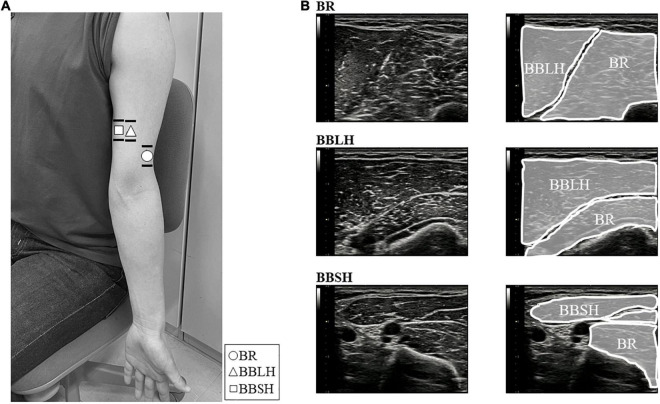
Images of the electrode locations and ultrasonogram for each muscle. **(A)** The location of surface (lines) and fine-wire (symbols) electrodes of the BR (○), BBLH (△), and BBSH (□). Surface electrodes were placed across the wire insertion of each muscle as shown in the horizontal lines. Fine-wire electrodes were inserted approximately where the symbols were shown. **(B)** The upper, middle, and lower panels represent the ultrasound images of the BR, BBLH, and BBSH muscles, respectively. BBLH, long head of the biceps brachii muscle; BBSH, short head of the biceps brachii muscle; BR, brachialis muscle.

Intramuscular and surface EMG were recorded from three elbow flexors: the BR, the long head of the BB (BBLH), and the short head of the BB (BBSH). Intramuscular EMG was measured using urethane-coated stainless steel, soft, fine-wire electrodes (0.03 mm; Unique Medical Inc., Tokyo, Japan) in bipolar configuration (5 mm inter-tip distance). Fine-wire electrodes placed in disposable 25-gauge hypodermic needles were inserted into each muscle by an experienced orthopedic surgeon (YN) under ultrasonographic guidance (SNiBLE, Konica Minolta Inc., Japan). The ultrasonography probe was transversely placed on the elbow flexors and the location of each muscle was visualized (Figure 1A). The electrode locations are shown and described in Figure 2 and Table 1, respectively. After insertion, we confirmed the electrode location within the muscles during elbow flexion by ultrasonography.

**TABLE 1:** Electrode locations for each muscle.

Muscle	Electrode location
BR	Distal part of the upper arm where the muscle becomes superficial (Staudenmann and Taube, 2015), approximately two to three fingerbreadths lateral to the line between acromion and cubital fossa, and without interference of the adjacent muscle (BBLH and triceps brachii muscle)
BBLH	Center of the muscle belly, on the line between medial acromion and cubital fossa cubit, approximately 1/3 proximal from the fossa cubit (Hermens et al., 2000), and without interference of the adjacent muscle (BBSH)
BBSH	Center of the muscle belly, two to three fingerbreadths medial to the line between acromion and cubital fossa, approximately 1/3 proximal from the cubital fossa (Hermens et al., 2000), and without interference of the adjacent muscles (BBLH and triceps brachii muscle)

*BBLH, long head of the biceps brachii muscle; BBSH, short head of the biceps brachii muscle; BR, brachialis muscle.*

Surface EMG was measured using Ag-AgCl disc electrodes (∅8 mm; Intercross Inc., Tokyo, Japan) with conductive paste in a bipolar configuration (20 mm inter-electrode distance). We confirmed the superficial area of the BR during the dynamic elbow flexion movement for each participant by ultrasonography (Figure 1B). To obtain the same or adjacent muscle fiber activity as that for intramuscular EMG, the surface electrodes were placed across the insertion of the fine-wire electrodes in a longitudinal direction over the muscles (Figure 2). Reference electrodes were attached to the skin over the lateral epicondyle of the elbow.

### Data Analysis

All EMG signals were recorded using a wireless EMG system (intercross-413, Intercross Inc., Tokyo, Japan). The signals were amplified (gain: 1,000), band-pass filtered (20–500 Hz), and recorded on a PC. The sampling frequency was 1,000 Hz.

The EMG data were analyzed using a MATLAB-based original program (R2020b, The MathWorks Inc., United States). To analyze the independent activity of each muscle, the differentiated EMG signals from the raw data were extracted for the middle 2 s of the MVICs task (Winter et al., 1994) and averaged for each muscle. The interval between the start and end time of elbow flexion was taken for the analysis of the elbow-flexion task. Elbow movements were detected using the radial and ulnar styloid process of their measured upper limb as anatomical bony markers, with the start and end times being identified based on the movements of these markers captured using a video camera. The start time was defined as the beginning of the movement from the resting limb position, and the end time was defined as the time when elbow joint was fully flexed and completely stopped. All EMG signals obtained during the isotonic elbow-flexion task were zero-lag band-pass filtered between 20–500 Hz (4th order, Butterworth). The filtered EMG signals were smoothened using a moving root-mean-square filter (time window: 300 ms), thereby computing the envelope of the signals (Farfán et al., 2010). Each EMG signal was time-normalized (1–101 frames) and averaged for each participant and muscle. The amplitudes of the signals were normalized to the peak activity of the task to decrease inter-individual variabilities (Peter et al., 2019).

### Statistical Analysis

The peak cross-correlation coefficients between the differentiated EMG signals were calculated using the MATLAB xcorr function. If the mean coefficients between muscles were lower than 0.3, then the independent of the muscles was established (Winter et al., 1994).

The EMG time-series of synergistic muscles are highly correlated and time dependent (Robinson et al., 2015). For the isotonic elbow-flexion task, to examine the time dependent differences in the EMG patterns between those obtained from the surface and fine-wire electrode, statistical parametric mapping (SPM) analysis was used. SPM analysis is a MATLAB-based software package for statistical analysis (spm1d).^1^ An SPM two-tailed non-parametric test was used to compare the surface and intramuscular EMG patterns. First, we calculated the scalar output statistic, SPM{t} (Adler and Taylor, 2007) to form a statistical non-parametric map. SPM{t} is a scalar trajectory variable that shows the magnitude of the differences between the surface and intramuscular EMG patterns. We then tested the null hypothesis by calculating the critical threshold at which only α% (set to 5%) of smooth random curves would be expected to traverse. This critical threshold calculation is based on estimates of trajectory smoothness via temporal gradients (Friston, 2007) and random field theory expectations regarding the field-wide maximum (Adler and Taylor, 2007). EMG time-series were considered significantly different if any values of SPM{t} exceeded the critical threshold.

Additionally, to examine the degree of similarity in the EMG patterns recorded by the surface electrodes and fine-wire electrodes for the three muscles, Pearson’s correlation coefficient between the electrodes for each muscle were determined (Jacobson et al., 1995). After normalizing the correlation coefficients as z-score by Fisher r-to-z transformation, the z-scores were compared using ANOVA with a factor of muscles (BR, BBLH, BBSH). *Post hoc* tests were performed using a Bonferroni’s test. For comparing the z-scores, SPSS version 23 statistical software (IBM Inc., Chicago, IL, United States) was used and statistical significance was set at a *p* < 0.05.

## Results

In the MVICs task, all cross-correlation coefficients between the surface EMG signals from the muscles were lower than 0.3 (BR–BBLH, 0.18 ± 0.05; BR–BBSH, 0.16 ± 0.03; BBLH–BBSH, 0.20 ± 0.06).

**FIGURE 3:**
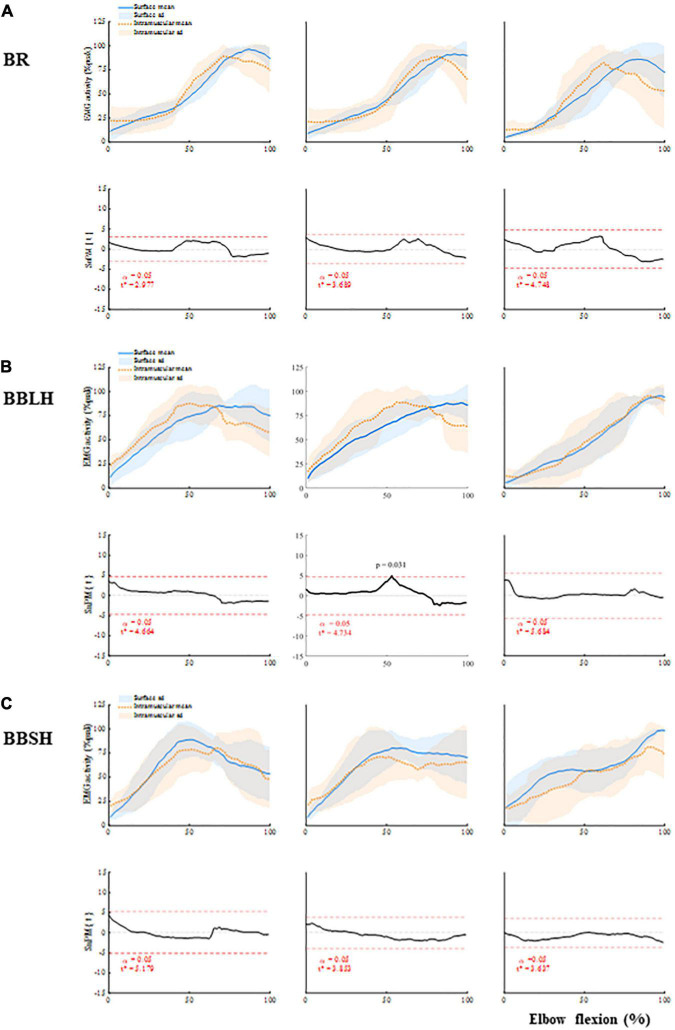
Muscle activity of the elbow flexors and comparisons between the surface and intramuscular EMG **(A)** BR, **(B)** BBLH, and **(C)** BBSH activities, and the comparisons between surface and intramuscular EMG. Upper panels of each muscle represent mean and standard deviation of the muscle activity for each forearm position. Lower panels of each muscle represent the scalar output statistic, SPM{t} (solid black lines), and the critical thresholds (t*) calculated for α significance level defining supra-threshold clusters for SPM{t} trajectories (dashed red lines). BBLH, long head of the biceps brachii muscle; BBSH, short head of the biceps brachii muscle; BR, brachialis muscle; EMG, electromyography; SPM, statistical parametric mapping.

In the isotonic elbow-flexion task, there were no significant differences between the surface and intramuscular EMG patterns of the BR activity in any of the forearm positions (Figure 3A). The EMG patterns of the BR activity were similar between the three forearm positions, showing a gradual increase in activity and peaking at approximately 70–90% of elbow flexion (Figure 3A).

There were also no significant differences between the surface and intramuscular EMG patterns of the BBLH and BBSH activities in any of the forearm positions (Figures 3B,C), except for the BBLH activity at the 54% of elbow flexion in neutral forearm position (*p* = 0.031). However, the EMG patterns of the BBLH and BBSH activities differed between the forearm positions. In the supinated and neutral forearm positions, the EMG patterns of the BBLH and BBSH activities showed an increase in activity from approximately 30–70% of elbow flexion and thereafter remained at the same level. In the pronated forearm position, the EMG patterns of the BBLH and BBSH activities showed a gradual increase in activity, peaking at approximately 80–100% of elbow flexion.

The r values of the EMG patterns between the surface and fine-wire electrodes for each muscle are shown in Supplementary Table 1. There was no significant main effect of the muscles in any of the forearm positions (supination: *F* = 1.1, *p* = 0.362; neutral: *F* = 4.0, *p* = 0.053; pronation: *F* = 1.8, *p* = 0.837).

## Discussion

In this study, we compared the EMG patterns of elbow flexor activities recorded using surface and fine-wire electrodes. Our results showed that the EMG patterns, including those of BR activity, were not significantly different between the surface and fine-wire electrodes. Additionally, the r values, which represented the degree of similarity of the EMG patterns between the surface and fine-wire electrodes, did not significantly differ between the BR and BB activity. These results indicate that surface EMG can be used to measure the BR activity as well as the BB activity. The cross-correlation coefficients between the BR and BB were < 0.3, suggesting that the recorded BR and BB activities were highly independent of each other (Winter et al., 1994).

We also validated the location of the elbow flexors under ultrasonographic guidance. Muscle geometry could be changed following a change in the elbow joint angle, and there is a possibility of shift of superficial area of the BR during dynamic elbow flexion movement. However, by using ultrasonography, we also confirmed that the fine-wire electrodes were located within the muscles and the location of the surface electrode were on the superficial area of the BR, even during elbow flexion. Therefore, each EMG signal represented the individual muscle activity of one of the elbow flexors.

We observed that the EMG patterns of the BR activity were similar among the different forearm positions, whereas those of the BB activities were not. It is noteworthy that the BR is inserted into the ulna, while the BB is inserted into the radius (Neumann, 2010). This anatomical difference can cause variations in the BB activity at different forearm positions, without affecting the BR activity (Basmajian and De Luca, 1985; Murray et al., 1995; Naito, 2004; Neumann, 2010).

Intramuscular EMG is necessary to assess the electrical activity of deep as well as small muscles; however, this method is highly invasive and does not represent the electrical activity of the whole muscle (Bogey et al., 2000). Contrastingly, while surface EMG has potential limitations such as crosstalk and difficulty in measuring the activities of deep muscles (Bogey et al., 2000), this method can record the electrical activity of a muscle over a large area, easily and non-invasively. Our results showed that if the elbow flexors are identified under ultrasonographic guidance, surface EMG can be a feasible method to record the BR activity.

This study includes some limitations. First, the number of participants was small. Intramuscular EMG studies are known to have a relatively small sample size due to the highly invasive nature of the study (Peter et al., 2019). Second, all participants in this study were healthy males with a thin subcutaneous fat layer. Excessive overlying adipose tissue can influence the myoelectric signals (Kuiken et al., 2003). Finally, the participants of this study performed the elbow flexion without lifting any weight like dumbbells. Since the size of EMG amplitude is strongly influenced by performed force levels, the results of this study are restricted to the tasks or exercises with minimal lifting of weight. For these reasons, our results are limited in their generalizability.

In conclusion, our results indicated that there were no differences between the surface and intramuscular EMG patterns of the BR activity during the elbow flexion that was done without lifting any weight, and the EMG patterns of the BR activity and the BB activity were independent of each other. Thus, surface EMG can be used an easier and non-invasive alternative for measuring the BR activity, which conventionally has been measured with intramuscular EMG.

## Data Availability Statement

The original contributions presented in the study are included in the article/Supplementary Material, further inquiries can be directed to the corresponding author.

## Ethics Statement

The studies involving human participants were reviewed and approved by the Research Ethics Committee of the Hiroshima University Hospital (Approval Number: C299). The patients/participants provided their written informed consent to participate in this study.

## Author Contributions

SD, HK, and TS conducted the literature review, conceived the study, and structured the study design. SD, YN, and TS were involved in the data acquisition. SD, HK, and YI were involved in obtaining the ethical approval. SD performed the data analysis. SD, HK, AU, KK, and TS contributed to the interpretation of the results and writing of the article. All authors read and approved the final manuscript.

## Conflict of Interest

The authors declare that the research was conducted in the absence of any commercial or financial relationships that could be construed as a potential conflict of interest.

## Publisher’s Note

All claims expressed in this article are solely those of the authors and do not necessarily represent those of their affiliated organizations, or those of the publisher, the editors and the reviewers. Any product that may be evaluated in this article, or claim that may be made by its manufacturer, is not guaranteed or endorsed by the publisher.

## Footnotes

1
https://spm1d.org

## Abbreviations

ANOVA: analysis of variance
BBLH: long head of the biceps brachii muscle
BBSH: short head of the biceps brachii muscle
BR: brachialis muscle
EMG: electromyography
MVIC: maximum voluntary isometric contraction
SPM: statistical parametric mapping.

## References

[B1] Adler R. J. Taylor J. (2007). *Random Fields and Geometry.* New York, NY: Springer.

[B2] An K. N. Hui F. C. Morrey B. F. Linscheid R. L. Chao E. Y. (1981). Muscles across the elbow joint: a biomechanical analysis. *J. Biomech.* 14 659–669. 10.1016/0021-9290(81)90048-8 7334026

[B3] Basmajian J. V. De Luca C. J. (1985). *Muscles Alive.* Baltimore, MD: Williams & Wilkins.

[B4] Basmajian J. V. Latif A. (1957). Integrated actions and functions of the chief flexors of the elbow: a detailed electromyographic analysis. *J. Bone Joint Surg. Am.* 39-A 1106–1118. 13475410

[B5] Bogey R. A. Perry J. Bontrager E. L. Gronley J. K. (2000). Comparison of across-subject EMG profiles using surface and multiple indwelling wire electrodes during gait. *J. Electromyogr. Kinesiol.* 10 255–259. 10.1016/s1050-6411(00)00015-8 10969199

[B6] Buchanan T. S. Almdale D. P. Lewis J. L. Rymer W. Z. (1986). Characteristics of synergic relations during isometric contractions of human elbow muscles. *J. Neurophysiol.* 56 1225–1241. 10.1152/jn.1986.56.5.1225 3794767

[B7] Farfán F. D. Politti J. C. Felice C. J. (2010). Evaluation of EMG processing techniques using Information Theory. *Biomed. Eng. Online* 9:72. 10.1186/1475-925X-9-72 21073705 PMC2989313

[B8] Friston K. (2007). *Statistical Parametric Mapping: The Analysis of Functional Brain Images.* Amsterdam: Elsevier/Academic Press.

[B9] Genet F. Schnitzler A. Droz-Bartholet F. Salga M. Tatu L. Debaud C. (2017). Successive motor nerve blocks to identify the muscles causing a spasticity pattern: example of the arm flexion pattern. *J. Anat.* 230 106–116. 10.1111/joa.12538 27595994 PMC5192805

[B10] Hermens H. J. Freriks B. Disselhorst-Klug C. Rau G. (2000). Development of recommendations for SEMG sensors and sensor placement procedures. *J. Electromyogr. Kinesiol.* 10 361–374. 10.1016/s1050-6411(00)00027-4 11018445

[B11] Hodges P. W. Pengel L. H. Herbert R. D. Gandevia S. C. (2003). Measurement of muscle contraction with ultrasound imaging. *Muscle Nerve* 27 682–692. 10.1002/mus.10375 12766979

[B12] Jacobson W. C. Gabel R. H. Brand R. A. (1995). Surface vs. fine-wire electrode ensemble-averaged signals during gait. *J. Electromyogr. Kinesiol.* 5 37–44. 10.1016/s1050-6411(99)80004-2 20719635

[B13] Kawakami Y. Nakazawa K. Fujimoto T. Nozaki D. Miyashita M. Fukunaga T. (1994). Specific tension of elbow flexor and extensor muscles based on magnetic resonance imaging. *Eur. J. Appl. Physiol. Occup. Physiol.* 68 139–147. 10.1007/BF00244027 8194543

[B14] Knox J. Gupta A. Banwell H. A. Matricciani L. Turner D. (2021). Comparison of EMG signal of the flexor hallucis longus recorded using surface and intramuscular electrodes during walking. *J. Electromyogr. Kinesiol.* 60:102574. 10.1016/j.jelekin.2021.102574 34273727

[B15] Kuiken T. A. Lowery M. M. Stoykov N. S. (2003). The effect of subcutaneous fat on myoelectric signal amplitude and cross-talk. *Prosthet. Orthot. Int.* 27 48–54. 10.3109/03093640309167976 12812327

[B16] Li L. Tong K. Y. Hu X. (2007). The effect of poststroke impairments on brachialis muscle architecture as measured by ultrasound. *Arch. Phys. Med. Rehabil.* 88 243–250. 10.1016/j.apmr.2006.11.013 17270524

[B17] Luca C. J. D. (1997). The use of surface electromyography in biomechanics. *J. Appl. Biomech.* 13 135–163.

[B18] Mackinnon S. E. Novak C. B. Myckatyn T. M. Tung T. H. (2005). Results of reinnervation of the biceps and brachialis muscles with a double fascicular transfer for elbow flexion. *J. Hand Surg. Am.* 30 978–985. 10.1016/j.jhsa.2005.05.014 16182054

[B19] Murray W. M. Buchanan T. S. Delp S. L. (2000). The isometric functional capacity of muscles that cross the elbow. *J. Biomech.* 33 943–952. 10.1016/s0021-9290(00)00051-8 10828324

[B20] Murray W. M. Delp S. L. Buchanan T. S. (1995). Variation of muscle moment arms with elbow and forearm position. *J. Biomech.* 28 513–525. 10.1016/0021-9290(94)00114-j 7775488

[B21] Naito A. (2004). Electrophysiological studies of muscles in the human upper limb: the biceps brachii. *Anat. Sci. Int.* 79 11–20. 10.1111/j.1447-073x.2004.00064.x 15088788

[B22] Neumann D. A. (2010). *Kinesiology of the Musculoskeletal System: Foundations for Rehabilitation.* St. Louis, MI: Mosby/Elsevier.

[B23] Onishi H. Yagi R. Akasaka K. Momose K. Ihashi K. Handa Y. (2000). Relationship between EMG signals and force in human vastus lateralis muscle using multiple bipolar wire electrodes. *J. Electromyogr. Kinesiol.* 10 59–67. 10.1016/s1050-6411(99)00020-6 10659450

[B24] Õunpuu S. Deluca P. A. Bell K. J. Davis R. B. (1997). Using surface electrodes for the evaluation of the rectus femoris, vastus medialis and vastus lateralis muscles in children with cerebral palsy. *Gait Post.* 5 211–216.

[B25] Perry J. Easterday C. S. Antonelli D. J. (1981). Surface versus intramuscular electrodes for electromyography of superficial and deep muscles. *Phys. Ther.* 61 7–15. 10.1093/ptj/61.1.7 7454803

[B26] Peter A. Andersson E. Hegyi A. Finni T. Tarassova O. Cronin N. (2019). Comparing surface and fine-wire electromyography activity of lower leg muscles at different walking speeds. *Front. Physiol.* 10:1283. 10.3389/fphys.2019.01283 31649557 PMC6796797

[B27] Robinson M. A. Vanrenterghem J. Pataky T. C. (2015). Statistical Parametric Mapping (SPM) for alpha-based statistical analyses of multi-muscle EMG time-series. *J. Electromyogr. Kinesiol.* 25 14–19. 10.1016/j.jelekin.2014.10.018 25465983

[B28] Rudroff T. Staudenmann D. Enoka R. M. (2008). Electromyographic measures of muscle activation and changes in muscle architecture of human elbow flexors during fatiguing contractions. *J. Appl. Physiol.* 104 1720–1726. 10.1152/japplphysiol.01058.2007 18356480

[B29] Staudenmann D. Taube W. (2015). Brachialis muscle activity can be assessed with surface electromyography. *J. Electromyogr. Kinesiol.* 25 199–204. 10.1016/j.jelekin.2014.11.003 25468488

[B30] Winter D. A. Fuglevand A. J. Archer S. E. (1994). Crosstalk in surface electromyography: theoretical and practical estimates. *J. Electromyogr. Kinesiol.* 4 15–26. 10.1016/1050-6411(94)90023-X 20870543

