# Supplementary material for: Brachialis Muscle Activity Can Be Measured With Surface Electromyography: A Comparative Study Using Surface and Fine-Wire Electrodes
Source: Front Physiol. 2021 Dec 23;12:809422. doi: 10.3389/fphys.2021.809422 (PMC8733609; doi:10.3389/fphys.2021.809422)
Supplement: Supplementary file 1 [file Table_1.docx]

***Supplementary Material***

Supplemental Table 1. R values of the EMG patterns between the surface and fine-wire electrodes

|  | BR | BBLH | BBSH |
| --- | --- | --- | --- |
| Supination | 0.88 ± 0.15 | 0.78 ± 0.18 | 0.83 ± 0.12 |
| Neutral | 0.91 ± 0.10 | 0.65 ± 0.22 | 0.85 ± 0.07 |
| Pronation | 0.84 ± 0.12 | 0.85 ± 0.07 | 0.65 ± 0.30 |
| Abbreviations: BBLH, long head of the biceps brachii muscle; BBSH, short head of the biceps brachii muscle; BR, brachialis muscle; EMG, electromyography | | | |
